# β-Secosterol, an Oxyphytosterol Produced Through the Reaction of β-Sitosterol with Ozone, Demonstrates Different Cytotoxic Effects on BRL-3A and HTC Cells

**DOI:** 10.3390/biom15070939

**Published:** 2025-06-27

**Authors:** Bianca S. Takayasu, Igor R. Martins, Miriam Uemi, Janice Onuki, Glaucia M. Machado-Santelli

**Affiliations:** 1 Department of Cell and Developmental Biology, Institute of Biomedical Sciences, University of São Paulo, São Paulo 05508-000, Brazil; bianca.takayasu@gmail.com; 2 Laboratory of Structural Biology, Butantan Institute, São Paulo 05503-900, Brazil; 3 Department of Chemistry, Institute of Environmental, Chemical and Pharmaceutical Sciences, Federal University of São Paulo, São Paulo 09972-270, Brazil; ig.rodmat@gmail.com (I.R.M.); 4 Center for Natural and Human Sciences, Federal University of ABC, São Paulo 09280-560, Brazil; miriam.uemi@ufabc.edu.br (M.U.)

**Keywords:** oxyphytosterols, sitosterol, β-secosterol, cytoskeleton, cell cycle, ozone

## Abstract

Sitosterol (Sito) is a phytosterol with bioactive properties, including reducing atherosclerosis risk and anti-inflammatory and antitumoral effects. However, it can be oxidized by reactive oxygen species such as ozone (O_3_), producing oxyphytosterols with harmful effects such as cytotoxicity, oxidative stress, and proatherogenicity. Ozone, a strong oxidant and common pollutant, can alter plant steroid compounds, raising concerns about dietary oxyphytosterol intake. Studies identify β-Secosterol (βSec) as the primary ozone-derived oxyphytosterol from Sito, exhibiting cytotoxic effects on HepG2 human liver tumor cells. This study investigated βSec’s biological effects on two rat liver cell lines: BRL-3A (immortalized) and HTC (tumoral), examining cell death, cell cycle progression, morphology, and cytoskeleton organization. While Sito influenced cell metabolic activity without affecting cell survival or morphology, βSec demonstrated significant cytotoxicity in both cell lines. It induced G0/G1 cell cycle arrest and disrupted cytoskeleton organization, with different implications: BRL-3A cells showed persistent cytoskeletal changes potentially linked to tumor induction, while HTC cells displayed chemoresistance, restoring cytoskeletal integrity and enhancing metastatic potential. These findings reveal βSec’s complex, context-dependent effects, suggesting it may promote tumor-like behavior in non-tumoral cells and resistance mechanisms in cancer cells, contributing to understanding oxyphytosterols’ implications for physiological and pathological conditions.

## 1. Introduction

**Figure 1:**
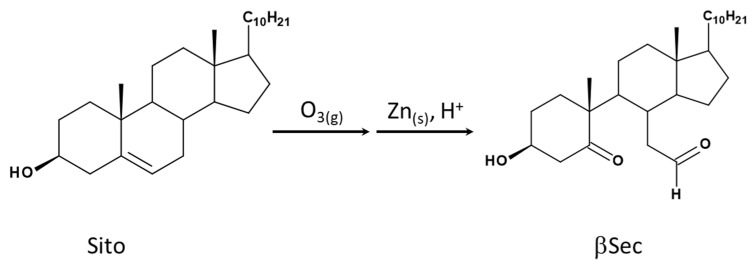
β-Secosterol (βSec) formation from Sitosterol (Sito) ozonization (for synthesis and characterization details, see Martins et al., 2020 [19]).

Phytosterols are steroid compounds with notable bioactive properties [1]. Among them, Sitosterol (Sito) (Figure 1) is one of the most abundant and widely distributed, commonly found in vegetable oils, seeds, grains, and fruits, as well as phytosterol-enriched functional foods and supplements [2]. Sito has gained attention for its ability to reduce the risk of atherosclerosis by inhibiting intestinal cholesterol absorption, thereby increasing the consumption of plant sterols [3]. Moreover, Sito has been employed as a chiral pool for synthesizing structurally modified bioactive derivatives with promising antitumoral [4] and cholesterol-lowering activities [5]. Beyond this, Sito and its derivatives exhibit various potential pharmacological properties, including anti-inflammatory, lipid-lowering, antiatherosclerotic, hepatoprotective, and antitumor effects [6], including in hepatocarcinoma [7].

However, despite these benefits, phytosterols are vulnerable to oxidation by reactive oxygen species such as ozone (O_3_), heat, or enzymatic activity, leading to the formation of oxyphytosterols [8]. Oxyphytosterols are associated with either beneficial or deleterious biological effects, including cytotoxicity, apoptosis [9], mitochondrial dysfunction [10], oxidative stress [11], proatherogenicity [3,11], modulation of inflammation [12], antiviral activity [13], and antiproliferative effects on various tumor cell lines [14].

Oxyphytosterols, some detectable in human plasma and tissues, may originate from dietary sources or endogenous formation, although the exact mechanisms of their in vivo production remain unclear [15]. Baumgartner et al. (2022) [16] hypothesized that phytosterol oxidation occurs primarily in the liver or plasma, followed by preferential hepatic elimination. This hypothesis is supported by elevated oxyphytosterol content in the liver tissue of mice with high plasma phytosterol concentration.

Ozone (O_3_), known for its strong oxidative potential, is widely employed as a sanitizing agent in the agri-food and food-processing industries [17]. Furthermore, rising O_3_ concentrations in the troposphere, particularly in urban areas, have raised agricultural and health concerns [18]. Excessive O_3_ exposure in plants can alter secondary metabolites, including steroid compounds, potentially disrupting several physiological processes. This may lead to reduced plant growth and yields and points out potential risks of adverse health effects due to increased dietary intake of phytosterols and oxyphytosterols [15].

Although no direct evidence of in vivo oxyphytosterol formation from ozone oxidation has been established, our group performed the synthesis and chemical characterization of ozone-derived oxyphytosterols and identified β-Secosterol (βSec) as the primary product from Sito ozonization [19] (Figure 1).

Our previous studies demonstrated that βSec reduces the viability of HepG2 tumor liver cells, induces morphological alterations through cytoskeleton disorganization, and causes cell cycle arrest in the G0/G1 phase [20]. Nevertheless, its specific role in the observed biological effects remains uncertain, with potential for both beneficial and harmful outcomes. Then, we considered it essential to conduct comparative studies across non-tumoral and tumoral cell lines, helping determine whether βSec selectively affects malignant processes and/or induces broader cytotoxic effects.

Therefore, this study evaluated the impact of the ozone-derived oxyphytosterol, βSec, on two rat liver-derived cell lines—BRL-3A (immortalized, nontumoral) and HTC (hepatoma)—by assessing cytotoxicity, cell death, cell cycle, morphology, and cytoskeleton organization, and explored their potential pathophysiological significance for tumor induction, chemoresistance, and metastasis.

## 2. Materials and Methods

### 2.1. Synthesis and Characterization of Oxyphytosterol βsec

Synthesis, purification, and chemical characterization of βSec, a Sitosterol seco aldehyde 2-[(7aR)-5-[(1R,4S)-4-hydroxy-1-methyl-2-oxocyclohexyl]-1,7a-dimethyl-1,2,3,3a,4,5,6,-octahydroinden-4-yl]acetaldehyde), were performed as described by Martins et al., 2020 [19]. Briefly, the oxidation of Sito (Sigma-Aldrich; St. Louis, MO, USA) occurred in a 24 mM solution of Sito in dichloromethane (CH_2_Cl_2_) at 4 °C under an ozone flow (90 mL/min) for 1.5 h. Then, the solvent was removed under reduced pressure and subsequently reduced with zinc in an acid medium. The organic phase was removed under reduced pressure. The oxyphytosterols formed in the reaction were purified by Thin Layer Chromatography (TLC) on a silica gel column using a hexane and ethyl acetate gradient.

### 2.2. Preparation of Compounds

The compounds Sito and βSec were previously dissolved in pure ethanol to obtain a final concentration of 10 mM. The stock solution was separated into aliquots and stored at −20 °C. A new aliquot was used in each experiment, and the final percentage of ethanol in the incubations was 0.5%.

### 2.3. Cell Maintenance and Cultivation

The BRL-3A (CRL-1442) and HTC (code 0112) cell lines from ATCC (Washington, DC, USA) and Rio de Janeiro Cell Bank (Rio de Janeiro, RJ, BR), respectively, were cultivated in DMEM F-12 (Sigma-Aldrich; St. Louis, MO, USA) supplemented with 10% fetal bovine serum (Cultilab; Campinas, SP, BR), 100 IU/mL penicillin, and 0.1 mg/mL streptomycin (Gibco; Grand Island, NY, USA) and maintained at 37 °C with atmosphere containing 5% CO_2_.

### 2.4. Cell Metabolic Activity—MTT Assay

BRL-3A or HTC cells were plated in 96-well microplates (5 × 10^3^ cells/well) and incubated overnight at 37 °C and 5% CO_2_. Cells were treated with Sito or βSec at 0.1 to 20 µM for 24 h, 48 h, and 72 h. Cell metabolic activity was evaluated after 4 h incubation with 100 µL/well of [3-(4,5-dimethylthiazol-2yl)-2,5-diphenyl tetrazolium] bromide (MTT 0.5 mg/mL), and formazan crystals were dissolved with 100 µL of pure DMSO per well [21]. Absorbance reading was performed in a spectrophotometer SpectraMax M5 Multi-Mode Microplate Reader (Molecular Devices; San Jose, CA, USA) at the reference wavelength of 570 nm, subtracting the background absorbance at 630 nm. Data were expressed as a percentage of cell metabolic activity relative to control. The concentration values that affect 50% of cell metabolic activity were calculated using the Quest Graph™ IC50 Calculator online software [22].

### 2.5. Cell Viability—Trypan Blue Exclusion Assay

BRL-3A cells (3.1 × 10^4^ cells/well) or HTC (5 × 10^4^ cells/well) were seeded in a 6-well plate, incubated for 48 h, and exposed to Sito (0.1 and 0.2 µM for BRL-3A; 0.2 and 0.5 µM for HTC) or βSec (2 and 4 µM for BRL-3A; 1 and 2 µM for HTC) for 48 h. Cells were collected by trypsinization (trypsin 0.05%), and an aliquot of each sample was stained with trypan blue (0.4%) [23]. Live and dead cells (trypan blue incorporation) were counted in a Neubauer chamber. The results were expressed as a percentage of cell viability relative to the control.

### 2.6. Cell Viability—Fluorescent Cell Staining with Hoechst 33342 and Propidium Iodide

BRL-3A and HTC cells were plated and treated under the same trypan blue exclusion assay conditions. After treatment, the plates were centrifuged at 244× *g* for 10 min, and 1.5 mL of the supernatant was removed, and 0.5 mL of the staining solution containing Hoechst 33342 and propidium iodide, previously prepared, was added to obtain a final concentration of 1 µg/mL of each dye [24]. The samples were incubated at 37 °C for 20 min and observed under a LionHeart FX fluorescence microscope from Biotek (Winooski, VT, USA). To construct the graphs, random fields were selected for manual counting of viable cells and mitosis (stained in blue by Hoechst 33342), dead cells (stained in red by propidium iodide and/or by merging with Hoechst 33342) until a count of at least 1 × 10^4^ total cells per sample was obtained.

### 2.7. Analysis of the Distribution of Cell Cycle Phases

For analysis of cell cycle phase distribution [25], BRL-3A cells (3.1 × 10^4^ cells/well) or HTC (5 × 10^4^ cells/well) were seeded in a 6-well plate, incubated until reaching the log phase of growth (48 h), and exposed to Sito (0.1 and 0.2 µM for BRL-3A; 0.2 and 0.5 µM for HTC) or βSec (2 and 4 µM for BRL-3A; 1 and 2 µM for HTC) for 48 h. The samples were fixed with 75% absolute methanol at 4 °C for 1 h and washed with phosphate-buffered saline (PBSA). DNA was stained with PBSA solution containing propidium iodide (10 µg/mL) and RNase (1 mg/mL) at 4 °C for 1 h and analyzed in a flow cytometer (GUAVA EasyCyte Plus, Hudson, MA, USA).

### 2.8. Analysis of Morphology and Cytoskeletal Organization

Cells were cultured on coverslips in 35 mm diameter plates and treated with Sito (0.2 µM for BRL-3A and 0.5 µM for HTC) or βSec (2 and 4 µM for BRL-3A and 1 and 2 µM for HTC) for 48 h. Cells were washed three times with PBSA (Phosphate Buffered Saline: 137 mM NaCl; 2.7 mM KCl, 10 mM Na_2_HPO_4_; 2 mM KH_2_PO_4_; pH 7.2–7.4) and fixed for 30 min with 3.7% formaldehyde, washed three times with PBSA, and permeabilized with Triton X-100 (0.5%) for 30 min [26]. After three washes with PBSA, the samples were immunostained with primary monoclonal antibodies produced in mice, anti-α-tubulin (1:50) and anti-β-tubulin (1:50) from Sigma-Aldrich (St. Louis, MO, USA), and incubated overnight. Corresponding secondary antibody anti-mouse IgG (H + L), F(ab’)_2_ Fragment (Alexa Fluor^®^ 488 Conjugate) (1:50) from Cell Signaling Technology^®^ (Danvers, MA, USA), was incubated for 2 h. Actin microfilaments were labeled with 2 h incubation with phalloidin conjugated to Alexa Fluor^®^ 555 (1:20) from Sigma-Aldrich (St. Louis, MO, USA). Subsequently, nuclei were labeled with DAPI (1:100) from Sigma-Aldrich (St. Louis, MO, USA), and the coverslips were mounted with Vecta-Shield (Vector Laboratories, Newark, CA, USA). Three washes with PBSA were performed between each incubation, and no blocking step was necessary. Analyses were performed on the LionHeart FX fluorescence microscope from Biotek (Winooski, VT, USA) and the TCS SP8 confocal microscope from Leica (Wetzlar, HE, Germany).

### 2.9. Analysis of Actin Filaments and Focal Adhesion

BRL-3A and HTC cells were subjected to the same plating, fixation (30 min with 3.7% formaldehyde), and permeabilization (Triton X-100 (0.5%) for 30 min) conditions described in the morphology and cytoskeletal organization analysis. We used 4 µM βSec for BRL-3A and 2 µM for HTC for 48 h. The cells were immunostained with a primary monoclonal antibody produced in rabbits, anti-vinculin (1:20) from Abcam (Cambridge, CAM, UK), and incubated overnight. To avoid nonspecific binding, we blocked the cells with a 1% BSA solution before immunostaining with the corresponding secondary antibody anti-rabbit IgG (H + L), fragment F (ab’)2 conjugated to Alexa Fluor^®^ 555 (1:50) from Cell Signaling Technology^®^ (Danvers, MA, USA), which was incubated for 2 h. Microfilaments were stained after 2 h incubation with phalloidin conjugated to Alexa Fluor^®^ 488 (1:50) from Sigma-Aldrich (St. Louis, MO, USA). Nuclei were labeled with DAPI (1:100) from Sigma-Aldrich (St. Louis, MO, USA), and coverslips were mounted with Vecta-Shield (Vector Laboratories, Newark, CA, USA). Between each incubation, three washes with PBSA were performed. Analyses were performed using a LionHeart FX fluorescence microscope from Biotek (Winooski, VT, USA).

### 2.10. Cell Recovery

The same treatment procedures were used for cell recovery evaluation to βSec effects (4 µM for BRL-3A and 2 µM for HTC) for 48 h. Still, after exposure to the compound, the supernatant was removed, and the samples were carefully washed with PBSA and incubated with 2 mL of DMEM F-12 medium for 48 h at 37 °C in an atmosphere containing 5% CO_2_. After this time, trypan blue exclusion analyses, distribution of cell cycle phases, morphological analysis, organization, and focal adhesion were performed according to the previously described procedures.

### 2.11. Statistical Analysis

Results were presented as mean ± SD or SEM of the percentage relative to the control of three independent experiments with three replicates (cell viability results) or three independent experiments performed in duplicate (cell cycle analysis). All data processing was performed using ANOVA and subsequent Dunnett tests for multiple comparisons with the control. Results were considered statistically significant when *p* < 0.05. For testing, we used GraphPad Prism software, version 8.0.1.

## 3. Results

### 3.1. Analysis of Cell Metabolic Activity and Cell Viability

**Figure 2:**
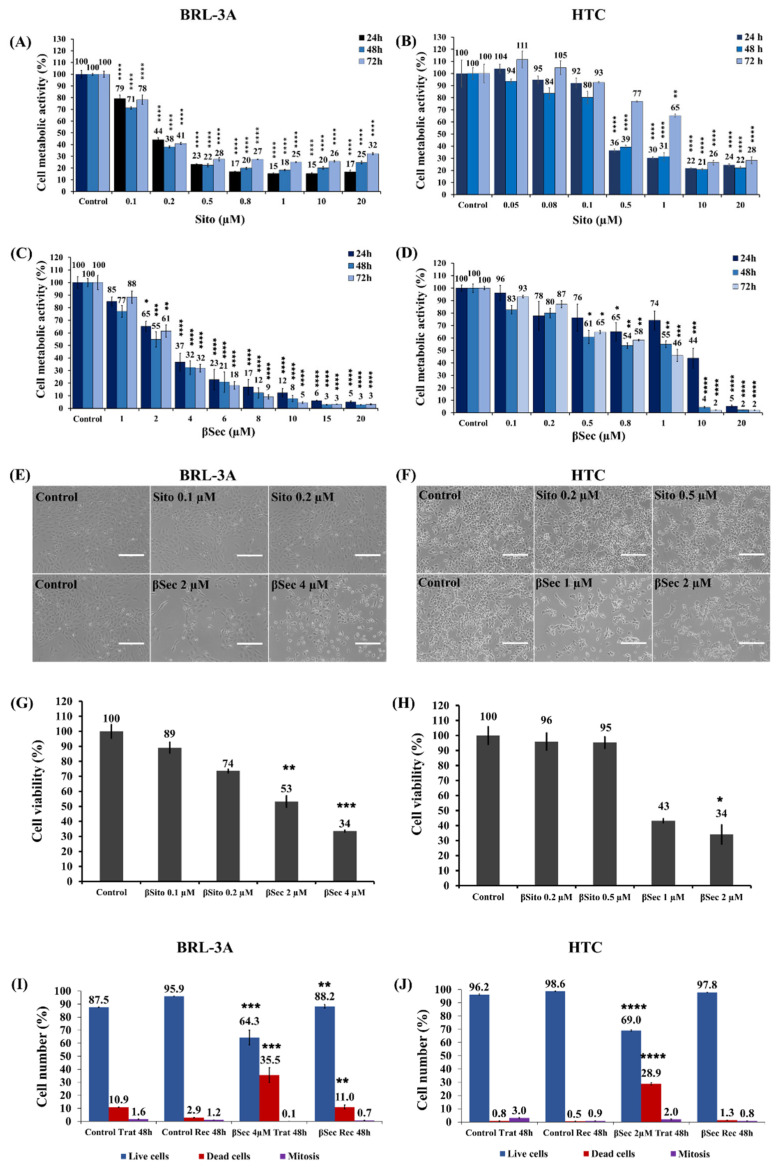
Cell metabolic activity and viability. Cell metabolic activity was measured by MTT assay, BRL-3A (**A**) and HTC (**B**) cells exposed to 0.05 µM to 20 µM Sito for 24 h, 48 h, and 72 h; BRL-3A (**C**) and HTC (**D**) cells exposed to 0.1 µM to 20 µM βSec for 24 h, 48 h, and 72 h. Data expressed as mean ± SEM of the relative percentage of the control. Statistical analysis was performed by ANOVA-Dunnett’s test for multiple comparisons versus the control (* *p* < 0.5, ** *p* < 0.01, *** *p* < 0.001, **** *p* < 0.0001). Observation of cell morphology in phase microscopy of BRL-3A (**E**) and HTC (**F**) cells and cell viability measured by trypan blue exclusion assay (**G** and **H**, respectively) after exposure of cells to βSito (0.1 and 0.2 µM for BRL-3A; 0.2 and 0.5 µM for HTC) and βSec (2 and 4 µM for BRL-3A; 1 and 2 µM for HTC) for 48 h. Counting of the number of live cells (Hoechst 33342), dead cells (propidium iodide), and mitosis rate after treatment for 48 h (**Trat**) and after 48 h recovery (**Rec**) of BRL-3A cells (**I**) to 4 μM βSec or HTC cells (**J**) to 2 μM βSec. Data are expressed as the mean ± SEM of the percentage relative to the control of three independent experiments with three replicates for each sample. Statistical analysis was performed using ANOVA-Dunnett’s test for multiple comparisons versus control (* *p* < 0.5, ** *p* < 0.01, *** *p* < 0.001, **** *p* < 0.0001). Scale bar: 200 µm (**E**,**F**).

Exposure of BRL-3A cells to Sito showed a concentration-dependent decrease in cell metabolic activity from 0.1 µM to 0.5 µM in 24 h (0.1 µM: 79.1 ± 5.6%, and 0.5 µM: 23.2 ± 0.9%), 48 h (0.1 µM: 71.3 ± 1.9%, and 0.5 µM: 22.5 ± 2.5%), and 72 h (0.1 µM: 78.3 ± 6.8%, and 0.5 µM: 27.6 ± 2.4%). Sito treatments reached a plateau from 0.8 to 20 µM, maintaining cell metabolic activity at around 20% in the three treatment times (Figure 2A). Similar results were observed in HTC cells. However, a significant decrease in cell metabolic activity occurred from 0.5 µM in 24 h (36.4 ± 2.3%) and 48 h (39.4 ± 2.8%). Plateau formation started from 1 µM at 24 and 48 h (Figure 2B).

In treatments with βSec, we observed a concentration- and time-dependent decrease in metabolic activity of BRL-3A cells with a significant reduction starting at 2 µM (65.2 ± 7.1% in 24 h; 54.6 ± 12.4% in 48 h; 61.5 ± 8.5% at 72 h) (Figure 2C). For HTC cells, there was a significant decrease in metabolic activity from 0.8 µM (64.9 ± 12.9% at 24 h; 53.9 ± 3.7% at 48 h; 58.3 ± 1.3% at 72 h) (Figure 2D), demonstrating lower resistance to the compound compared to BRL-3A. However, in both cell lines, viable cells were almost completely reduced by 20 µM of βSec in the three treatment times.

From the MTT assay, we calculated the concentrations of Sito and βSec that affected 50% of cell metabolic activity for both cell lines to be used in the next experiments.

Despite the decrease in cell metabolic activity measured by the MTT assay, Sito did not reduce cell density, as observed under bright field microscopy (Figure 2E,F), or induce cell death, as measured by the trypan blue exclusion assay in both cell lines (Figure 2G,H). Conversely, βSec treatment decreased cell density and the number of viable cells (Figure 2E–H).

To investigate if βSec effects are reversible, we performed the LIVE/DEAD assay using fluorescent staining with Hoechst 33342 (HO) and propidium iodide (PI). BRL-3A cells treated with βSec 4 µM showed a significant decrease in the number of live cells, from 87.5 ± 0.2% of the control to 64.3 ± 21.7%, along with an increase in the percentage of cell death (35.5 ± 21.8%) compared to the control (10.9 ± 0.3%) (Figure 2I). The rate of mitosis did not show significant differences between treatment and control. After removing the compound, we observed recovery of BRL-3A cells, with a live cell percentage of 88.2 ± 2.3% compared to 95.9 ± 0.4% of the control.

Exposure of HTC cells to 2 µM βSec (Figure 2J) caused a significant decrease in live cells, with a percentage of 69.0 ± 1.5% compared to 96.2 ± 0.9% of the control. We also observed a considerable increase in the rate of cell death, from 0.8 ± 0.3% of the control to 28.9 ± 1.6% of the treated cells. HTC cells showed recovery capability after the removal of βSec for 48 h, presenting a live cell percentage of 97.8 ± 0.2%, along with a decrease in the percentage of cell death (1.3 ± 0.2%) compared to the 48-h treatment (28.9 ± 1.6%). No significant differences were observed in the rate of mitosis compared to the control.

### 3.2. Analysis of the Cell Cycle

**Figure 3:**
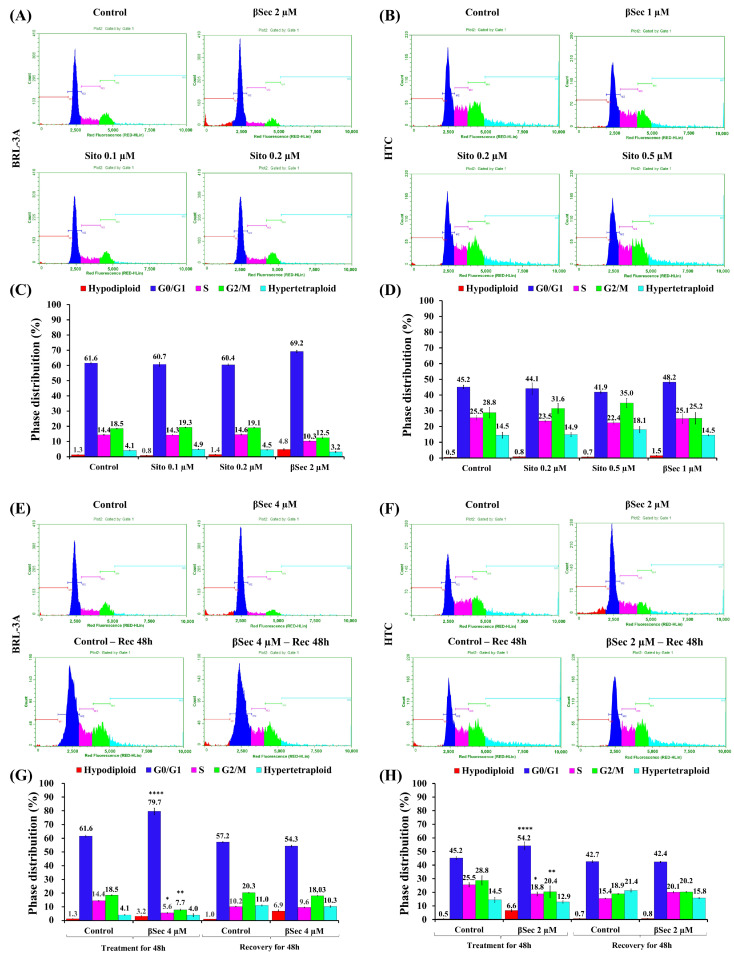
Distribution of cell cycle phases. Flow cytometry distribution of cell cycle phases of BRL-3A (**A**,**C**) and HTC cells (**B**,**D**) after treatment with Sito (0.1 and 0.2 µM for BRL-3A; 0.2 and 0.5 µM for HTC) and βSec (2 µM for BRL-3A; 1 µM for HTC) for 48 h. BRL-3A (4 µM) (**E**,**G**) and HTC (2 µM) (**F**,**H**) cells were treated with βSec for 48 h and recovered for 48 h. Data are presented as the mean ± SEM of the relative percentage compared to the control, based on three independent experiments performed in duplicate for each sample. Statistical analysis was conducted using ANOVA-Dunnett’s test for multiple comparisons vs. control (* *p* < 0.05, ** *p* < 0.01, **** *p* < 0.0001).

Flow cytometry analysis of cell cycle progression showed that Sito treatment did not interfere with cell distribution in the cell cycle phases in both cell lines (Figure 3A–D). Differently, BRL-3A cells exposed to 4 µM of βSec for 48 h (Figure 3E,G) showed an increase in the G0/G1 phase, with 79.7 ± 8.5%, when compared to 61.6 ± 2.7% of the control, and a consequent decrease in S phase (5.6 ± 1.3%) and G2/M (7.7 ± 2.4%) compared to control (S phase: 14.4 ± 1.2% and G2/M phase: 18.5 ± 1.1%). Similarly, treatments for 48 h with 2 µM of βSec (Figure 3F,H) also increased the cell population in the G0/G1 phase of HTC cells (54.2 ± 4.9%) when compared to control (45.2 ± 1.9%) and decreased from 25.5 ± 2.7% (control) to 18.8 ± 2.5% in the S phase cell population and from 28.8 ± 5.9% (control) to 20.4 ± 7.5% in the G2/M. Therefore, βSec causes cell cycle arrest in the G0/G1 phase in both cell lines, and for BRL-3A, it was necessary to double the concentration of βSec (4 µM) to cause the same effect observed in HTC tumor cells (2 µM).

To determine whether the effects of βSec on the cell cycle in both cell lines are reversible, we analyzed the distribution of cell cycle phases following a 48-h removal of oxyphytosterol exposure. BRL-3A and HTC cells exhibited a similar distribution of cell cycle phases, suggesting a capacity for cell cycle progression recovery once the compound was removed (Figure 3G,H).

### 3.3. Analysis of Cell Morphology and Cytoskeletal Organization

**Figure 4:**
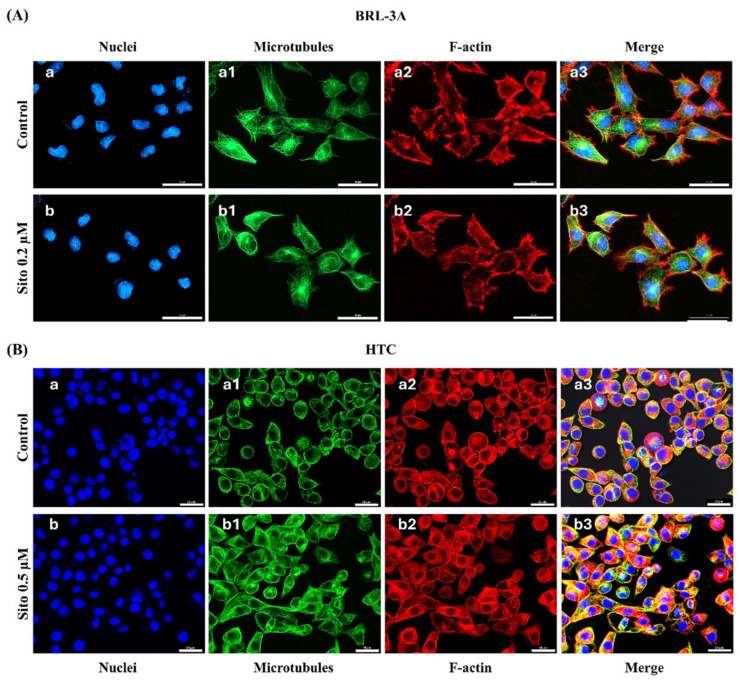
Morphology and cytoskeletal organization of BRL-3A (**A**) and HTC (**B**) cells. Fluorescence microscopy images of control (**a**–**a3**) and after 48 h treatment (**b**–**b3**) with Sito 0.2 µM (BRL-3A) and 0.5 µM (HTC), submitted to immunofluorescence reaction with antibodies against α and β-tubulin plus anti-mouse secondary antibodies conjugated to Alexa Fluor 488 (green). Microfilaments of f-actin were stained with phalloidin conjugated to Alexa Fluor 555 (red). Nuclei were stained with DAPI (blue). Scale bar: 30 µm (**A**); 25 µm (**B**).

The potential effects of the compounds on cell morphology were analyzed in immunofluorescence preparations with anti-tubulin antibodies and phalloidin. Exposure to Sito 0.2 µM for 48 h did not modify the cytoskeleton distribution or nuclei morphology of BRL-3A cells compared to the control, with cells maintaining their characteristic epithelial morphology (Figure 4A). Likewise, HTC cells exposed to Sito 0.5 µM for 48 h did not show morphological changes compared to the control (Figure 4B).

**Figure 5:**
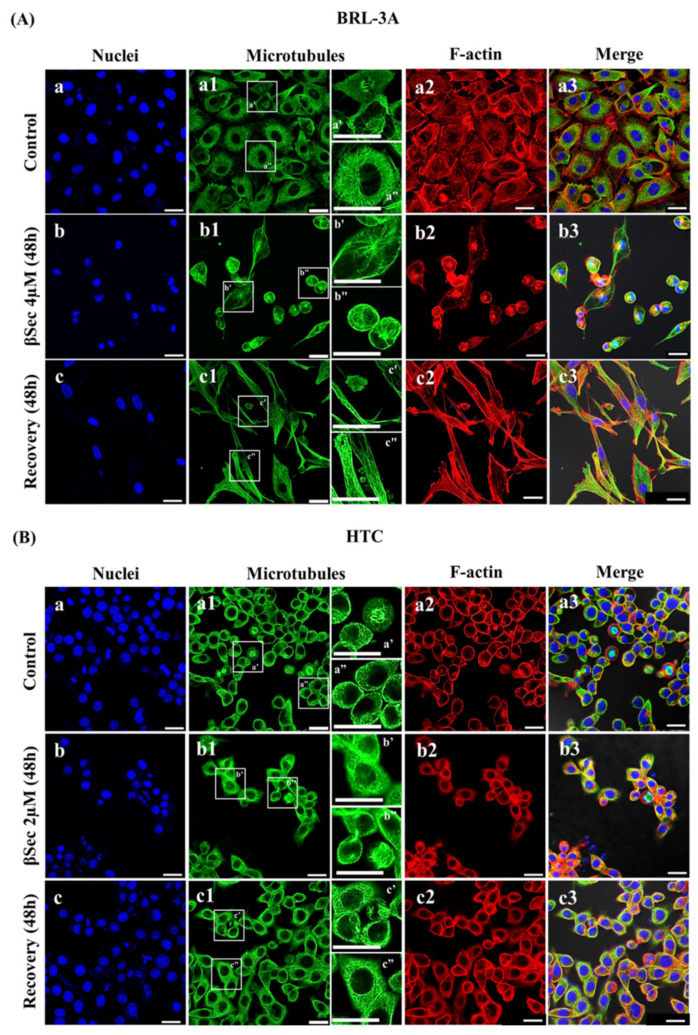
Morphology and cytoskeleton organization of BRL-3A (**A**) and HTC (**B**) cells. Fluorescence microscopy images of control (**a**–**a3**,**a’**,**a”**), after 48 h of treatment with 2 or 4 µM βSec (**b**–**b3**,**b’**,**b”**), and after 48 h of recovery (**c**–**c3**,**c’**,**c”**). Cells were subjected to immunofluorescence staining with antibodies against α- and β-tubulin, plus secondary anti-mouse antibodies conjugated to Alexa Fluor 488 (green). F-actin filaments were stained with phalloidin conjugated to Alexa Fluor 555 (red), and nuclei were stained with DAPI (blue). Scale bar: 25 µm.

Conversely, BRL-3A cells (Figure 5A) exposed to 4 µM of βSec for 48 h showed modifications in morphology, with rounded cells and more densely stained microtubules at the cell periphery, indicating reorganization of the microtubule network (Figure 5A(b1,b’,b”,b3)). We also observed disorganization of the microfilaments, revealed by the loss of stress fibers and the formation of actin aggregates near the nucleus (Figure 5A(b2,b3)). These results indicate that βSec could interfere with the cytoskeleton dynamics of BRL-3A cells. When HTC cells (Figure 5B) were treated with βSec 2 µM for 48 h, there were alterations in the microtubule and microfilament networks, showing reorganization with a more diffuse distribution of α- and β-tubulin dimers (Figure 5B(b1,b’,b”,b3)). Unlike in BRL-3A cells, βSec did not cause the formation of F-actin aggregates in HTC cells (Figure 5B(b2,b3)).

To investigate whether βSec acts irreversibly on the morphology and cytoskeleton of the two lineages, after treating the cells with oxyphytosterol for 48 h, the medium was changed to a normal, compound-free one for an additional 48 h. For BRL-3A cells, after removing the compound for 48 h, we observed an apparent increase in cell density and number of mitotic cells (Figure 5A(c3)) compared to the treated one (Figure 5A(b3)). However, cell morphology does not return to its initial appearance, with a more elongated and thicker cytoskeleton (Figure 5A(c1–c3)) than the control (Figure 5A(a1–a3)).

After removing βSec for 48 h from HTC cells, a considerable increase in cell density was observed (Figure 5B(c3)). The distribution profile of microtubule and actin networks (Figure 5B(c1,c2)) appears to recover its initial structure, similar to the control (Figure 5B(a1,a2)), but with more elongated cells (Figure 5B(c3)).

Furthermore, after removing the compound, analysis of the distribution of microtubule networks alone highlighted an altered organization of microtubules, presenting more defined filaments and the presence of rounded and clustered structures between the BRL-3A cells (Figure 5A(c’,c”)), which may suggest a death process. Conversely, HTC cells recovered the morphology after 48 h of βSec removal (Figure 5B(c’,c”)).

### 3.4. Focal Adhesion

**Figure 6:**
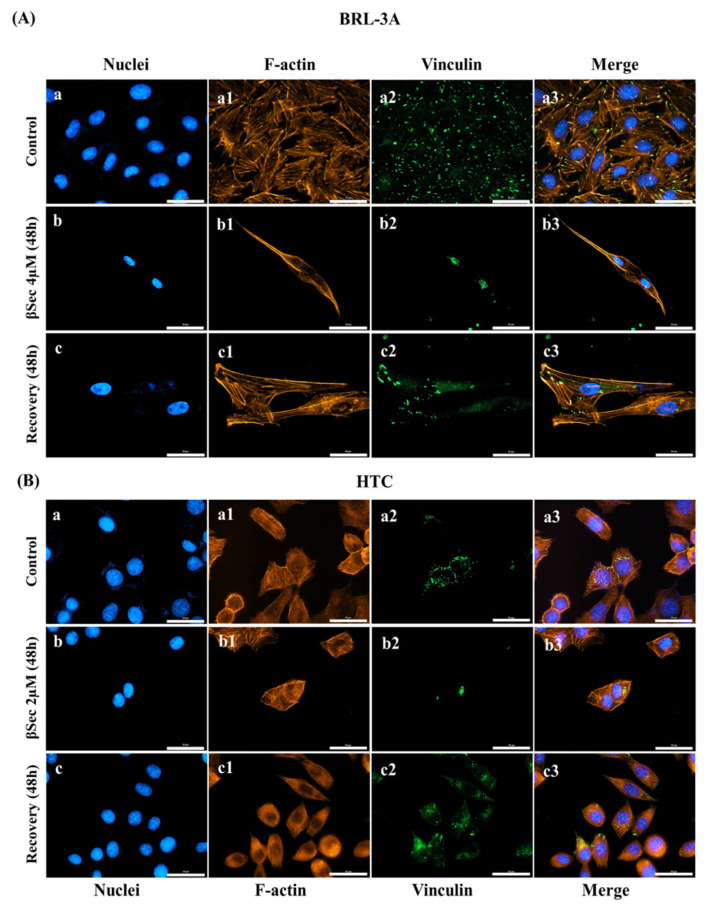
Analysis of focal adhesion in BRL-3A and HTC cells. Analysis of focal adhesion in BRL-3A (**A**) and HTC (**B**) cells. Fluorescence microscopy images of control (**a**–**a3**), after 48 h of treatment with βSec 4 µM for BRL-3A and 2 µM for HTC (**b**–**b3**), and after 48 h of recovery (**c**–**c3**). Cells were subjected to immunofluorescence staining using antibodies against vinculin, a protein localized at focal adhesion plaques, followed by Alexa Fluor 488 conjugated anti-rabbit secondary antibodies (green). F-actin filaments were stained with phalloidin conjugated to Alexa Fluor 555 (orange). Nuclei were stained with DAPI (blue). Scale bar: 30 µm.

Control BRL-3A cells showed vinculin predominantly distributed at the cell periphery and the ends of stress fibers formed by the microfilaments (Figure 6A(a1–a3)). The exposure of the cells to 4 µM βSec for 48 h showed differences in vinculin distribution, being found in the cytoplasmic region and near the nucleus, along with changes in actin filament distribution (Figure 6A(b1–b3)). After βSec removal for 48 h, we observed the recovery of vinculin distribution at the cell periphery, along with increased stress fibers of the microfilament network (Figure 6A(c1–c3)). However, BRL-3A cells did not recover their morphology after the removal of compound exposure, showing elongated and fusiform cells (Figure 6A(c3)).

HTC cells exposed to 2 µM βSec for 48 h (Figure 6B(b1–b3)) showed morphological changes with loss of vinculin distribution, which appeared in clumps (Figure 6B(b2)). However, after the compound’s removal, the cells recovered the distribution pattern of actin filaments and vinculin in focal adhesion points (Figure 6B(c1–c3)). They returned to a morphology similar to that of controls, with the usual distribution of vinculin (Figure 6B(c3)).

## 4. Discussion

β-Secosterol, a recently identified oxyphytosterol from Sito ozonization by our group [19], remains largely biologically uncharacterized. Thus, investigating βSec’s effects is crucial to understanding whether it contributes to the beneficial effects associated with Sito or whether it may promote toxicity.

Most studies regarding the hepatic antitumoral properties of Sito and oxyphytosterols have focused exclusively on tumor cell lines, such as HepG2, Huh-7, and HCCLM3 [7,20,27,28], underscoring the need for broader experimental designs that include non-tumor models. This is essential to evaluating the selectivity and safety of potential therapeutic agents.

Due to experimental limitations, using normal human hepatocytes remains challenging, primarily because of their limited availability, short lifespan in culture, and donor-dependent variability. Consequently, the BRL-3A and HTC rat liver cell lines are widely recognized and validated models, supporting comparative evaluation of compound effects on non-tumoral (BRL-3A) and tumoral (HTC) hepatocytes while preserving essential hepatic functions, which enables the investigation of metabolic and cytotoxic responses under physiological and pathological conditions [29]. Moreover, their high reproducibility, ease of culture, and ethical advantages make them a suitable alternative to in vivo liver models. In addition, these cell lines are particularly relevant for studying oxyphytosterols, as phytosterol oxidation primarily occurs in the liver or plasma, followed by preferential hepatic elimination in the murine model [16].

Both Sito and βSec affected the enzymatic metabolic activity of BRL-3A and HTC cells (Figure 2). Low concentrations of Sito (0.1 and 0.5 μM) influenced cellular metabolism, likely via effects on mitochondria, as indicated by decreased cell metabolic activity in the MTT assay, which mainly reflects mitochondrial dehydrogenase activity, rather than cell death [30]. Even at 40 μM, Sito did not induce cell death, changes in cell cycle distribution, or cytoskeletal organization.

In contrast, βSec exhibited cytotoxic effects in both cell lines, inducing cell death, as confirmed by trypan blue exclusion (Figure 2G,H) and by Hoechst 33342 and propidium iodide staining (Figure 2I,J). These findings underscore the distinct mechanisms and biological effects of Sito and βSec.

βSec induced G0/G1 cell cycle arrest in both BRL-3A and HTC cell lines (Figure 3G,H), but with potentially distinct functional outcomes in non-tumoral versus tumoral contexts. This cell cycle arrest has also been observed in MCF-7 human breast cancer cells following 24-h exposure to the oxyphytosterol 5α,6α-epoxysitosterol [31]. Cell cycle progression is regulated by tightly controlled mechanisms that safeguard genomic integrity [32]. Entry into the G1 phase is mediated by cyclin D, which activates cyclin-dependent kinases CDK4 and CDK6 [33]. Inhibition of CDK activity can halt this process, leading cells into a quiescent (G0) state. Given this, βSec may interfere with cyclin D expression or CDK4/6 activity, disrupting the G1/S transition [34]. Additionally, G1 arrest can result from DNA damage by activating the MRN complex (MRE11-RAD50-NBS1), which initiates ATM kinase signaling. ATM subsequently phosphorylates CHK2 and p53, inducing the CDK inhibitor p21, thereby preventing entry into S phase [32]. These mechanisms suggest that βSec may activate DNA damage response pathways or directly impair cell cycle regulators to induce arrest. Furthermore, cyclin D1 complexes act in cytoskeletal organization, cell adhesion, and motility [34]. Cyclin D1 deficiency in macrophages alters microfilaments, resulting in more rounded cells, similar to what was observed in BRL-3A cells (Figure 5A(b2,b3)), as well as impaired focal adhesion formation and reduced cell motility [35]. These findings support the hypothesis that βSec may interfere with cyclin D expression and/or activity.

In BRL-3A cells, G0/G1 cell cycle arrest might serve as a protective mechanism, enabling cells to survive cytotoxic stress, re-enter the cell cycle, and proliferate after βSec exposure is discontinued. Nevertheless, since the results showed no recovery of cytoskeleton organization, we hypothesized that βSec could modify the migration ability of BRL-3A cells, favoring their transformation, probably acting more as a pro-tumorigenic agent than an antitumoral one. In HTC tumoral cells, this arrest could enhance chemoresistance, allowing dormant cells to evade cytotoxic effects, re-enter the cell cycle, and contribute to metastasis [36,37]. Recovery of HTC cells after βSec exposure further supports this resistance mechanism.

The recovery responses diverged while βSec-induced cytoskeletal disorganization was observed in both cell lines. In BRL-3A cells, βSec treatment altered morphology, including an elongated and thickened cytoskeleton, budding structures, and giant cells after recovery (Figure 6A(c3) and Figure S1). In contrast, HTC cells restored their original cytoskeletal structure, suggesting a more effective recovery mechanism, which contributed to their resistance to cytotoxic stress, enhanced adaptability, and potential for metastasis [38].

Our findings also highlight the potential role of βSec in promoting anastasis, a process in which cells recover from apoptosis and regain functionality, contributing to malignancy and resistance to therapy [39]. Furthermore, cytoskeleton reorganization is intrinsically related to epithelial-mesenchymal transition (EMT), where epithelial cancer cells lose their tight junctions, become more motile, and acquire mesenchymal characteristics, supporting cell migration and metastasis [40].

In addition to cytoskeletal remodeling, vinculin redistribution was a prominent feature of βSec-treated cells. Vinculin, a focal adhesion protein, is critical in linking the actin cytoskeleton to the extracellular matrix (ECM) via integrins [41]. βSec treatment altered vinculin localization, concentrating it near the nucleus in BRL-3A cells (Figure 6A(b2)) and in a single region in HTC cells (Figure 6B(b2)), deviating from the uniform distribution observed in controls. These changes disrupted focal adhesion and cytoskeletal integrity, impairing cell adhesion and motility. Notably, upon βSec removal, vinculin distribution and actin organization normalized, demonstrating the reversibility of its effects (Figure 6A(c3),B(c3)).

Importantly, the reversibility of cytoskeletal disruption and vinculin distribution in HTC cells upon βSec removal suggests that it may act as a reversible modulator of microtubule-associated signaling pathways rather than a direct tubulin degradation or irreversible chemical modification agent.

Therefore, βSec may modulate the activity of RhoGTPases, such as Rho, Rac, and Cdc42, which act as molecular switches regulating signaling pathways that control actin filament organization, formation of structures such as lamellipodia and filopodia, and microtubule dynamics [42]. As central regulators of cell morphology, motility, proliferation, adhesion, and polarity, dysregulated RhoGTPase activities [43] could underlie βSec-induced cellular adaptations, potentially contributing to resistance and enhanced survival under cytotoxic conditions. Cell detachment and focal adhesion loss may result from the suppression of focal adhesion kinase (FAK)/Src signaling, which coordinates integrin-mediated adhesion and cytoskeletal dynamics [44].

The chemical structure of βSec suggests a possible relationship with its cytotoxic properties. Secosterol aldehydes, such as 5,6-secosterol A and B, are electrophilic oxidation products of cholesterol that contain reactive aldehyde and hydroxyl groups [45]. As is well known, aldehyde confers the ability to form covalent adducts with nucleophilic residues on proteins, primarily via Schiff base formation. Such modifications alter protein hydrophobicity, promote misfolding and aggregation, and can disrupt normal cellular processes [46,47].

Importantly, these structural interactions also extend to membrane components. Secosterols have been shown to react with aminophospholipids such as phosphatidylethanolamine, forming covalent adducts that reduce membrane fluidity and stability [48,49,50]. These membrane alterations may compromise cellular homeostasis, enhance membrane permeability, and affect receptor and enzyme functions embedded in lipid bilayers.

Altogether, these structural characteristics of βSec—particularly the electrophilic aldehyde group—may contribute both to its cytotoxic effects and the induction of stress responses that underlie the chemoresistance process. Therefore, the differential membrane reactivity and protein modification potential of βSec are plausible mechanisms linking its structure to the observed cellular phenotypes.

## 5. Conclusions

Although limited to in vitro assays using two rat liver cell lines, which could differ from in vivo conditions, our study demonstrates that βSec induces cytotoxicity, disrupts cytoskeletal organization, and modulates cell cycle progression. In non-tumoral BRL-3A cells, the effects may suggest a potential pro-tumoral outcome. Conversely, HTC hepatoma cells exhibited signs of resistance, including partial recovery from cytoskeletal and cell cycle disturbances, hallmarks commonly associated with metastatic potential and tumor recurrence. Collectively, these findings point to a potentially deleterious role for βSec rather than a protective one. This highlights the need for further investigation into its molecular targets and mechanisms of action to elucidate the physiological and pathological implications of this novel oxyphytosterol.

## Data Availability

The original contributions presented in this study are included in the article/Supplementary Materials. Further inquiries can be directed to the corresponding authors.

## Supplementary Materials

The following supporting information can be downloaded at: https://www.mdpi.com/article/10.3390/biom15070939/s1, Figure S1: 3D reconstruction and orthogonal view images from confocal microscopy after 48 h of recovery. Images of BRL-3A cells were obtained from a 3D reconstruction of 22 slices using confocal microscopy after 48 h of recovery following βSec exposure (Rec 48 h). (A) The top plane of the image and (B) the bottom plane of the same image. (C) Stacked images projection and ortogonal sections. Arrows indicate rounded cells. Cells were subjected to immunofluorescence staining with antibodies against α- and β-tubulin, followed by Alexa Fluor 488-conjugated anti-mouse secondary antibodies (green). F-actin filaments were stained with phalloidin conjugated to Alexa Fluor 555 (red), and nuclei were stained with DAPI (blue). Scale bar: 50 µm.
