# Supplementary material for: β-Secosterol, an Oxyphytosterol Produced Through the Reaction of β-Sitosterol with Ozone, Demonstrates Different Cytotoxic Effects on BRL-3A and HTC Cells
Source: Biomolecules. 2025 Jun 27;15(7):939. doi: 10.3390/biom15070939 (PMC12292371; doi:10.3390/biom15070939)
Supplement: Supplementary file 1 [file biomolecules-15-00939-s001.zip › biomolecules-3601122-supplementary.pdf]

Supplementary Figure:

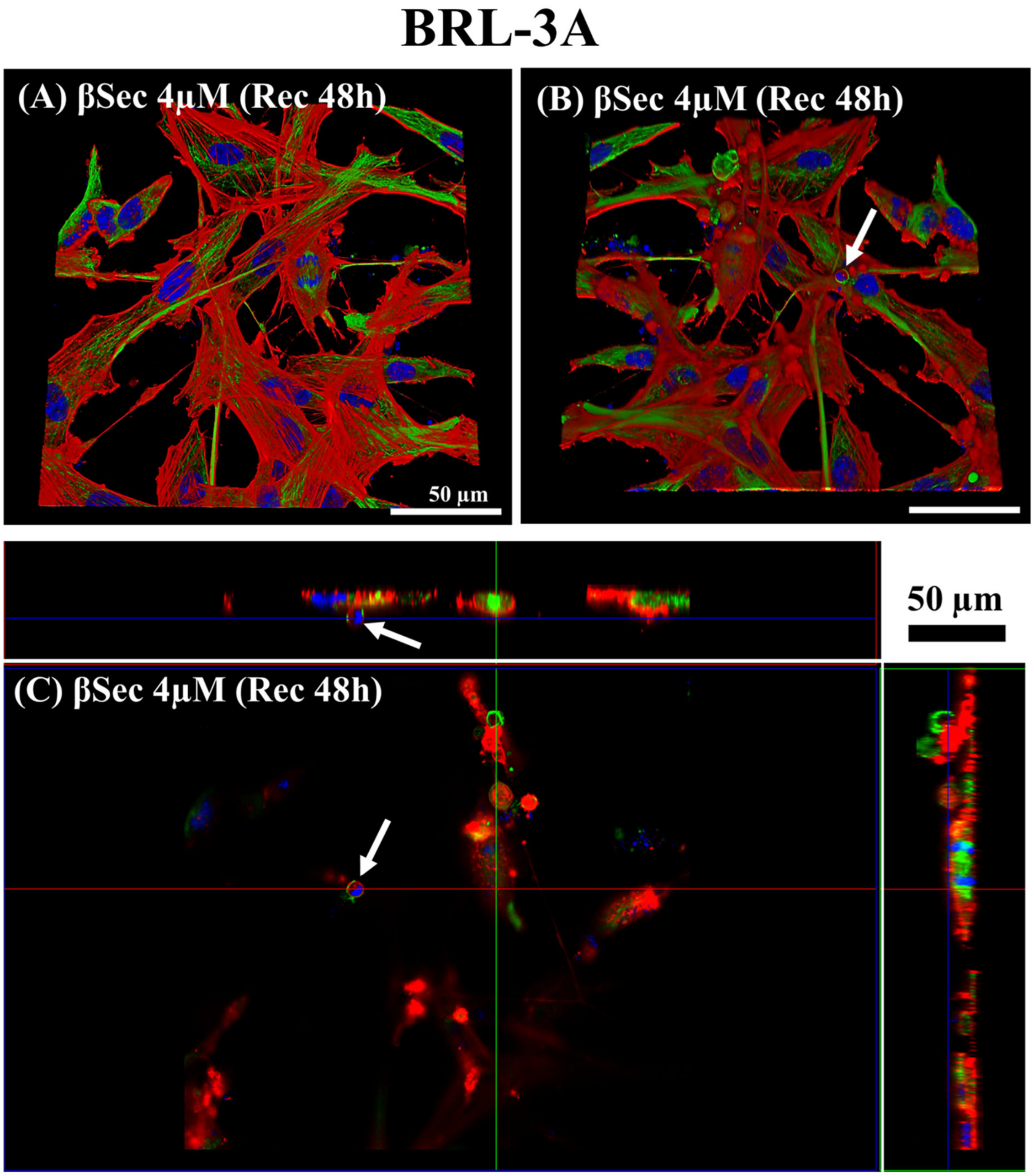

Figure S1. 3D reconstruction and orthogonal view images from confocal microscopy after 48 h of recovery. Images of BRL-3A cells were obtained from a 3D reconstruction of 22 slices using confocal microscopy after 48 h of recovery following βSec exposure (Rec 48 h). (A) The top plane of the image and (B) the bottom plane of the same image. (C) Stacked images projection and ortogonal sections. Arrows indicate rounded cells. Cells were subjected to immunofluorescence staining with antibodies against α- and β-tubulin, followed by Alexa Fluor 488-conjugated anti-mouse secondary antibodies (green). F-actin filaments were stained with phalloidin conjugated to Alexa Fluor 555 (red), and nuclei were stained with DAPI (blue). Scale bar: 50 μm.
